# Genetic Dissection and Quantitative Trait Loci Mapping of Agronomic and Fodder Quality Traits in Sorghum Under Different Water Regimes

**DOI:** 10.3389/fpls.2022.810632

**Published:** 2022-02-17

**Authors:** Vinutha K. Somegowda, Kodukula V. S. V. Prasad, Jalaja Naravula, Anilkumar Vemula, Sivasubramani Selvanayagam, Abhishek Rathore, Chris S. Jones, Rajeev Gupta, Santosh P. Deshpande

**Affiliations:** ^1^International Crops Research Institute for the Semi-arid Tropics-HQ, Patancheru, India; ^2^Department of Biotechnology, Vignan University, Vadlamudi, India; ^3^International Livestock Research Institute (ILRI), International Crops Research Institute for the Semi-arid Tropics Campus, Patancheru, India

**Keywords:** dry biomass, digestibility, drought, fodder quality, QTL, Sorghum

## Abstract

Livestock provides an additional source of income for marginal cropping farmers, but crop residues that are used as a main source of animal feed are characteristically low in digestibility and protein content. This reduces the potential livestock product yield and quality. The key trait, which influences the quality and the cost of animal feed, is digestibility. In this study, we demonstrate that sorghum breeding can be directed to achieve genetic gains for both fodder biomass and digestibility without any trade-offs. The genotypic variance has shown significant differences for biomass across years (13,035 in 2016 and 3,395 in 2017) while *in vitro* organic matter digestibility (IVOMD) showed significant genotypic variation in 2016 (0.253) under drought. A range of agronomic and fodder quality traits was found to vary significantly in the population within both the control and drought conditions and across both years of the study. There was significant genotypic variance (σg^2^) and genotypic × treatment variance (σgxt^2^) in dry matter production in a recombinant inbred line (RIL) population in both study years, while there was only significant σg^2^ and σgxt^2^ in IVOMD under the control conditions. There was no significant correlation identified between biomass and digestibility traits under the control conditions, but there was a positive correlation under drought. However, a negative relation was observed between digestibility and grain yield under the control conditions, while there was no significant correlation under drought population, which was genotyped using the genotyping-by-sequencing (GBS) technique, and 1,141 informative single nucleotide polymorphism (SNP) markers were identified. A linkage map was constructed, and a total of 294 quantitative trait loci (QTLs) were detected, with 534 epistatic interactions, across all of the traits under study. QTL for the agronomic traits fresh and dry weight, together with plant height, mapped on to the linkage group (LG) 7, while QTL for IVOMD mapped on to LG1, 2, and 8. A number of genes previously reported to play a role in nitrogen metabolism and cell wall-related functions were found to be associated with these QTL.

## Introduction

Sorghum is the fifth most important cereal crop grown globally, referred to as the great millet; it is cultivated in the semiarid regions of the world. The production of sorghum grain in India is 4,633 k metric tons (2020).^1^ However, biomass production data are scarce. The green fodder production demand in 2020 is 1,134 million tonnes, while dry fodder is 630 million tonnes with a deficit of 64.21 and 24.81%, respectively; additionally, crude protein effect of 26.52% and total digestible nutrients of 23.70% were reported.^2^ India has the largest livestock population globally, with a predicted demand for green and dry fodder, reaching 1,012 and 631 million metric tons, respectively, by the year 2050 (IGFRI-ICAR, 2013). However, there is competition for land mainly from increased urbanization and other commercial activities, resulting in the increased utilization of marginal land for agriculture.

Farmers in the semiarid regions with annual rainfall of around 700–800 mm or less are dependent on both agriculture produce and livestock to support their livelihoods (Blümmel et al., 2009). Crop residues are mainly used to feed livestock; however, most of these are currently derived from crop cultivars that have been extensively bred and selected for grain production, resulting in lower fodder quality in terms of nutrition and digestibility (Blümmel et al., 2010). Therefore, to meet the demand of fodder, tapping into the natural genetic variation and introducing a paradigm shift in breeding objectives to food-feed products with improved biomass quality has high importance for farmers. The key traits used for the assessment of food-feed crops, after the grain yield, are biomass yield and digestibility, usually measured as *in vitro* organic matter digestibility (IVOMD). A difference of 5% units (47 vs. 52%) in IVOMD, which was highly correlated with stover pricing, has been shown to be associated with a price premium of 20% and higher (Blümmel et al., 2013, 2014). Hence, breeding strategies for developing a dual-purpose sorghum crop, with a focused approach toward fodder quality, are considered more beneficial than focusing on the grain alone.

The current advances in biotechnology, especially new generation sequencing platforms with lower costs, have had a pivotal role in crop improvement, helping plant breeders achieve greater genetic gains (Desai et al., 2020). Most agronomically relevant traits and quality traits are quantitative in nature and therefore, are controlled by multiple genes. Thus, genes/loci governing these traits can be analyzed using quantitative trait locus (QTL) analysis or genome-wide association studies (GWASs) (Mace and Jordan, 2011; Miao et al., 2020). The availability of 732.2 MB of finished sorghum genome sequence in the public databases^3^ has been embraced by many researchers to identify further genes/QTL responsible for drought resistance and agronomically important traits (Zou et al., 2012; Mace et al., 2012, 2019; Higgins et al., 2014). Twelve QTLs for drought-associated traits [nodal root angle, root and shoot dry weight (DW), and total leaf area] were identified by Mace et al. (2012). A QTL analysis was performed on a RIL population with a total of 57 major QTLs detected for eight agronomically important traits by Zou et al. (2012). A total of 91 QTL for agronomic traits, including plant height, number of leaves and days to flowering, were identified by Reddy et al. (2013). Fine mapping of stay-green QTL on linkage group (LG) 10 (Kiranmayee et al., 2020) and the introgression of stay green QTLs for concomitant improvement of food and fodder traits have also been reported (Blümmel et al., 2015).

Over the past 20 years of research in sorghum, 220 traits have been studied, and 6,000 QTL have been reported, and further to this, a Sorghum QTL Atlas has been developed on a high resolution open platform to facilitate candidate gene identification (Mace et al., 2019). Despite the similarity of target traits to address the challenges faced by the researchers for second-generation biofuel and fodder quality improvement (Somegowda et al., 2021), funding has been skewed toward the development of biofuels. Here, we present a study conducted to assess a recombinant inbred line (RIL) population, selected from a preliminary study conducted to identify the contrasting parents for IVOMD and dry stalk yield (Somegowda et al., 2020) with the objectives to: (i) evaluate the performance of the RIL population under drought and control conditions over 2 years and (ii) identify QTLs and further analyze the loci and genomic regions associated with fodder quality.

## Materials and Methods

### Trial Location

Trials were conducted over 2 years during the post-rainy season, (from the month of November to March, during 2016-2017 and 2017-2018) in vertisol soils at ICRISAT, HQ, located at an altitude of 545 m above the mean sea level with latitude of 17.53° N and longitude of 78.27° E. The timing of the trials was designed to enable the assessment of the drought treatment, which is commonly observed in sorghum cultivation during this season. The seasonal weather parameters indicate the temperature (maximum and minimum), rainfall, and sunshine hours during the crop growth period (Supplementary Figure 1).

### Plant Material

The RIL population was developed following a plant × plant crossing of two lines, ICSV1 (a poor parent for IVOMD) and ICSV700 (a superior parent for IVOMD) (Somegowda et al., 2020). ICSV1 is a dual-purpose open pollinated variety (OPV) bred at ICRISAT-HQ following the pedigree breeding method (ICRISAT, 1995). ICSV700 is a dual purpose breeding line developed at ICRISAT in 1992. The confirmed selfed F_1_-plant was advanced to F_2_, and progenies were advanced to F_6_ following a modified Single Seed Decent method. The F_6_ progenies were self-bulked, and a set of 319 RIL individuals was generated for further use. The trial consisted of 330 entries, comprising 319 RILs, two parents (repeated four times each), and three check varieties. The checks (M 35-1, SPV1411, and Phule Vasudha) were all popular varieties grown during the post-rainy season in India.

### Experimental Design

The trials were laid in an Alpha Lattice design, with 11 entries per block and 30 blocks per replication. The trials were planted under two water regimes *viz*., drought (irrigation withheld), and control (well irrigated), with three replications each, evaluated over the 2 years.

### Agronomic Practices

A total set of 330 entries were planted following a randomized design. Sowing was performed with a tractor mounted four cone planter (John Deer, 7100 US model). Plants were sown in 2-meter row lengths at a spacing of 60 cm between rows and 10 cm between plants. A basal dose of ammonium phosphate @150 kg/ha was applied at planting. Plots were maintained following standard crop cultivation practices with no dffierences between the control and drought plots, except for irrigation.

### Drought Induction

Irrigation was withheld in the plots before initiation of booting, at 50 Days After Sowing (DAS) and maintained until maturity, whereas, in control plots, irrigation was provided following a regular schedule, i.e., around 1 h for every 15 days until harvesting. Furthermore, to avoid water seepage from the control to drought plots a buffer (eight rows of bulk sorghum) was established between the two treatment plots.

### Agronomic Data

The following data were recorded in field, days to fifty percent flowering (DFL-days), plant height (PH-cm), fresh weight (FW-kg per plot), DW (DW-kg per plot), and grain weight (GW-kg per plot).

### Fodder Quality Data

Harvested fodder was dried, chopped, and grounded (to allow the powder to pass through 1-mm sieve). Samples were scanned using Near-Infrared Spectroscopy (NIRS) on a FOSS Forage Analyzer 5000 (FOSS Instrumentation XDS RCA, Win ISI Chemometrics package IV, Denmark) with software package Win ISI II (version 1.5, Intra Soft International, LLC) and specific sorghum calibration equations, developed and validated by conventional laboratory analysis (Blümmel et al., 2010). The following parameters were assessed, nitrogen on a dry matter basis (NDM-%), acid and neutral detergent fiber (ADF and NDF-%), acid detergent lignin (ADL-%), metabolizable energy (ME-MJ/kg DM), and IVOMD.

### Statistical Analysis

Combined analysis of variance across years and treatments was performed using the Restricted Maximum Likelihood (REML) procedure of GenStat v20.1 edition for Windows (VSN International, 2020), considering year, treatment, and replication as fixed, block, and entry as random factors. In order to pool the data across years and treatments, individual year and treatment variances were modeled to error distribution using REML. BLUEs (Best Linear Unbiased Estimates for fixed effects) and BLUP’s (Best Linear Unbiased Predictors—for random effect terms) were estimated from the combined analysis of variance and mean comparisons were calculated between population mean and the means of the parents. Year-wise Karl Pearson’s correlation was performed to establish a relationship between all agronomic and NIRS traits under both of the treatments.

### Genotyping the RIL Population

DNA was isolated from leaves of 12-day-old seedlings using a modified hexadecyltrimethylammonium bromide (CTAB) protocol (Mace et al., 2003), and genotyping by sequencing (GBS) was performed using the *Ape*KI restriction enzyme used for complexity reduction (Elshire et al., 2011). SNPs were called using the TASSEL v5.2 GBS pipeline against the sorghum assembly v3.1 with default parameters. Raw SNP data were produced on 321 individuals (from the population and both parents) with 292,960 SNP markers identified. Filtering was carried out to identify monomorphic loci and SNP markers with >10% missing values, and minor allele frequencies <5% were excluded from further analysis. From these results, genotypes contributing more than 16% of missing values were removed. Thus, the total population size used for final analysis consisted of 314 genotypes with 1,141 SNP markers spanning across the ten chromosomes.

### Linkage Maps, QTL Analysis, and Annotations

The genotype file was converted into numeric format using the R package, GAPIT (Genome Association and Prediction Integrated Tool). The MAP function of QTL IciMapping software version 4.2 (Wang et al., 2014)^4^ was used to generate a linkage map. The three general steps involved in linkage map construction are: Grouping, performed on a basis of a threshold value of LOD score 5, while grouping LG wise, markers falling in different groups were removed, and then ordering was carried out using k-Optimality by LOD of 2-OptMAP with NN intials, with nnTwoOpt algorithm, followed by Rippling using SALOD (Sum of Adjacent LOD scores). The QTL map was generated using Composite Interval Mapping (CIM), which combines interval mapping with multiple marker regression analysis, which controls the effects of QTL on other intervals or LGs onto the QTL that is being tested, and thus increases the precision of QTL detection. The QTL analysis was performed using the BIP function of QTL IciMapping software version 4.2.53. The mapping method adapted for the analysis was the Inclusive Composite Interval Mapping of ADDitive QTL (ICIM-ADD) with 1,000 permutation runs for each trait and Inclusive Composite Interval Mapping of digenic EPIstatic QTL (ICIM-EPI) with LOD value of 3.MapChart software was used to visualize the final charts with locus and position (Voorrips, 2002).

### Identification of Genomic Regions and Annotations

The QTL and SNP identified from sorghum population (RIL and Reference set, respectively) were searched for orthologs’ orthologous regions in maize genome using an in-house Python-based script. The input files used were the Sorghum bicolor genome sequence (Sbicolor_454_v3.01.1.fa.gz), the annotation file (Sbicolor_454_v3.1.1.gene.gff3), the CDS (Sbicolor_454_v3.1.1.cds_primaryTranscript Only.fa) from the Phytozome database and the annotation file of Zea mays (Zmays_284_Ensembl-18_2010-01-MaizeSequence.gene.gff3) from the Ensemble database. For QTLs, since their size was relatively long (in million bases), the orthologous regions were deduced based on the orthology and synteny relationship of the (sorghum) genes located within the QTL boundaries against the maize genome. The gene orthology data were obtained by running OrthoFinder tool on sorghum and maize proteomes. On the other hand, for SNPs about a fifty-nucleotide-long flanking region on each side was extracted, and searched for a homologous regions in maize genome using NCBI-BLASTN tool (Evalue: 1E-05). The visualization was performed using Circos (Krzywinski et al., 2009).

## Results

### Genetic Variation Across Years and Within Treatments

There was a wide range of variation in all agronomic and fodder quality traits identified in both the years (Table 1). Flowering during 2016 was delayed (80 Days After Sowing—DAS), while flowering started early in 2017 (65 DASs); mean plant height was higher in 2017 (95.65 cm) compared to 2016 (73.73 cm). Variation in fresh and DWs was greater in 2016 than in 2017. For the fodder quality traits, the variation in nitrogen was greater in 2016 than in 2017 (Table 1). The fiber fractions and lignin content were lower in 2016, but the variation in the digestibility-related traits (ME and IVOMD) was low in the individual years. All agronomic traits, except GW, showed significant differences under both the drought and control treatments, while significant differences were only observed for fodder quality traits (nitrogen, fiber fractions, lignin, metabolizable energy, and IVOMD) under the drought treatment (Table 2).

**TABLE 1:** Range, genotypic variances, and genotype by treatment variances (ANOVA) for 2 years and across two treatments.

	Individual years		Year wise across two treatments				Treatment wise across two years.	
Treatment	2016	2017	2016		2017		Control	Drought
				
Traits	Range	Range	σ g^2^	σ gxt^2^	σ g^2^	σ gxt^2^	σ g^2^	σ g^2^
DFL (days)	80.93–111.30	66–111.14	20.23**	18.26**	32.24**	4.84**	19.89**	21.06**
PH (cm)	73.73–232.35	95.65–233.13	681.92**	414.15**	946.92**	44.03**	566.77**	803.26**
FW (kg/plot)	460.67–2120.62	356.39–1567.47	160332**	39301**	25730**	25069**	38777**	23925*
DW (kg/plot)	302.28–901.80	245.36–648.88	13035**	5471**	3395**	2057**	0.3*	2202*
GW (kg/plot)	281.53–540.81	201.77–481.26	1482**	2659**	1375**	3450**	1090*	0.01
NDM (%)	0.68–1.28	0.41–0.58	0.0007*	0.00393	–	0.002	0.01	0.01*
NDF (%)	55.41–61.73	66.64–70.73	0.784	0.979	0.03	0.388	-0.01	0.69**
ADF (%)	38.71–44.51	42.74–45.48	0.309	1.074	0.06	0.125	-0.07	0.49**
ADL (%)	4.07–4.83	4.84–5.23	0.007*	0.02635	0.0018	0.0049	0.01	0.01*
ME (MJ/kg DM)	6.52–7.33	6.94–7.33	0.007*	0.02703	–	0.0033	-0.01	0.02**
IVOMD(%)	45.58–50.16	47.91–50.06	0.253*	0.965	0.16	0.148*	-0.11	0.38**

DFL, days to fifty percent flowering; PH, plant height; FW, fresh weight; DW, dry weight; GW, grain weight; NDM, nitrogen on a dry matter basis; ADF and NDF, acid and neutral detergent fiber; ADL, acid detergent lignin; ME, metabolizable energy; IVOMD, in vitro organic matter digestibility; σg^2^, genotypic variances; σg × t^2^, genotype by treatment variances; *significant at p < 0.05; **significant at p < 0.01.

**TABLE 2:** Mean comparisons between contrasting parents and population under both the treatments for each year.

Year	2016						2017					
	
Treatment	Control			Drought			Control			Drought		
Traits	P1 vs P2	Pop vs P1	Pop vs P2	P1 vs P2	Pop vs P1	Pop vs P2	P1 vs P2	Pop vs P1	Pop vs P2	P1 vs P2	Pop vs P1	Pop vs P2
DFL (days)	0.99	5.13**	6.13**	3.85**	1.60**	5.46**	9.76**	−7.07**	2.69	11.86**	−7.11**	4.75**
PH (cm)	2.61	−1.05	1.55	−4.41	−54.83**	−59.24**	22.39**	−49.05**	−26.66**	7.89*	−36.55**	−28.66**
FW (kg/plot)	464.04**	−1294.50**	−830.46**	359.14**	−610.57**	−251.43*	263.07	−257.29**	5.78	299.72**	−143.21**	156.52**
DW (kg/plot)	100.55	−359.88**	−259.33**	163.34**	−169.28**	−5.95*	95.55*	−78.19*	17.36	147.89**	−106.22**	41.68**
GW (kg/plot)	−51.00	−83.30*	−134.30**	95.72**	49.93	145.65**	−94.97**	70.90**	−24.07	−159.62**	106.88**	−52.74**
NDM (%)	0.04**	0.02	0.06**	−0.04	0.03	−0.005	0.01	0.005	0.01	−0.02	−0.001	−0.02
NDF (%)	−3.87**	1.88	−1.99	−1.06	1.32	0.27	−1.31	0.83	−0.48	−0.50	0.78	0.28
ADF (%)	−3.77**	1.96	−1.80	−1.28**	1.35**	0.08	−1.53	0.71	−0.82	−1.01	0.76	−0.25
ADL (%)	−0.39**	0.17**	−0.22**	−0.20**	0.22**	0.01	−0.33	0.21	−0.13	−0.21	0.18	−0.03
ME (MJ/kg DM)	0.48**	−0.30	0.18**	0.15**	−0.24	−0.09**	0.29	−0.16	0.13	0.12	−0.11	0.01
IVOMD (%)	2.48**	−1.27**	1.21**	0.69	−1.30**	−0.61**	1.67*	−0.92	0.76	0.65	−0.67	−0.02

DFL, days to fifty percent flowering; PH, plant height; FW, fresh weight; DW, dry weight; GW, grain weight; NDM, nitrogen on a dry matter basis; ADF and NDF, acid and neutral detergent fiber; ADL, acid detergent lignin; ME, metabolizable energy; IVOMD, in vitro organic matter digestibility; σg^2^, genotypic variances; σg × t^2^, genotype by treatment variances; *significant at p < 0.05; **significant at p < 0.01, P1: ICSV 1; P2: ICSV 700; Pop: population.

### Genotypic and Treatment Effects

The results for trait range, genotypic variance (σg^2^), genotypic × treatment variance (σg **×** t^2^) for years, and treatment are presented in Table 1. The genotype and treatment variation for agronomic traits were significantly different between 2016 and 2017. However, this was not Cywinski the case for the fodder quality traits. Of the fodder quality traits, significant genotypic variance was identified for ADL, ME, and IVOMD in 2016 and for IVOMD in 2017.

### Correlation

The Pearson’s correlation for agronomic and fodder quality traits for 2016 and 2017 is presented in Tables 3a,b, respectively. Plant height was significantly correlated with the stover fresh (0.34 in 2016 and 0.60 in 2017) and dry (0.32 in 2016 and 0.58 in 2017) weights under control conditions. Under drought conditions, a correlation with stover fresh weight (0.41 in 2016 and 0.47 in 2017) was recorded in both years. The stover fresh and DWs were highly correlated with each other in both treatments and years, 0.85 (control) and 0.88 (drought), respectively, in 2016 and 0.88 (control) and 0.91 (drought) in 2017. Furthermore, the plant height showed significant positive correlation with both fresh and DW (0.41 and 0.36 in 2016 and 0.47 and 0.50 in 2017). For the fodder quality traits, significant positive correlations were observed in both treatments and years between the fiber fractions and lignin content. In 2016, fiber fractions had a strong positive correlation with lignin (0.81 control) and (0.88 drought), respectively, while the correlation was 0.76 (control) and 0.67 (drought) in 2017. Conversely, significant negative relations were observed between digestibility and the fiber fractions and lignin, under both control and drought treatments in both years. Nitrogen content had a significant negative relation with fiber fraction under both years and treatments. The nitrogen content and plant height also showed negative correlation of -0.34 and -0.10 in year 2016 under control and drought, while -0.29 and -0.15 significantly negatively correlated in year 2017. No significant correlation was found for any traits with GW or flowering in either treatment or years. Similarly, no significant correlation was found between agronomic and fodder quality traits.

**TABLE 3:** Correlation of agronomic and fodder quality under drought, (3a) for year 2016 (3b) for year 2017, diagonally; the upper side represents control, and the lower side is drought.

	DFL (days)	PH (cm)	FW (kg/plot)	DW (kg/plot)	GW (kg/plot)	NDM (%)	NDF (%)	ADF (%)	ADL (%)	ME (MJ/kg DM)	IVOMD (%)
**(3a) 2016**											
DFL (days)		0.08	0.06	−0.10	−0.06	0.19**	−0.32**	−0.24**	−0.11	0.23**	0.26**
PH (cm)	0.17**		0.85**	0.02	0.34**	−0.17*	0.03	−0.03	−0.04	0.12*	0.08
FW (kg/plot)	0.21**	0.88**		0.07	0.32**	−0.17*	0.05	0.00	−0.02	0.09	0.05
DW (kg/plot)	−0.01	0.10	0.18*		0.001	−0.05	0.16*	0.19**	0.13	−0.18*	−0.21**
GW (kg/plot)	0.09	0.41**	0.36**	0.05		−0.10	0.18*	0.26**	0.20	−0.13*	−0.18*
NDM (%)	−0.11*	−0.27**	−0.24**	−0.09	−0.34**		−0.46**	0.15*	0.29	−0.27**	−0.17*
NDF (%)	−0.20**	−0.01	−0.03	−0.01	0.07	−0.03		0.63**	0.39	−0.57**	−0.60**
ADF (%)	−0.23**	−0.07	−0.08	−0.02	−0.05	0.20**	0.81**		0.81**	−0.89**	−0.91**
ADL (%)	−0.27**	−0.13*	−0.14*	−0.02	−0.12*	0.29**	0.68**	0.84**		−0.79**	−0.78**
ME (MJ/kg DM)	0.20**	0.17*	0.16*	0.02	0.21**	−0.42**	−0.68**	−0.89**	−0.81**		0.96**
IVOMD (%)	0.19**	0.11*	0.11	0.02	0.14*	−0.31**	−0.72**	−0.90**	−0.81**	0.98**	
**(3b) 2017**											
DFL (days)		0.25**	0.22**	−0.08	0.25**	−0.06	−0.01	−0.08	−0.02	0.10	0.04
PH (cm)	0.31**		0.91**	0.04	0.60**	−0.23**	0.00	0.01	0.04	0.02	−0.02
FW (kg/plot)	0.30**	0.88**		0.05	0.58**	−0.17*	0.02	0.04	0.06	0.00	−0.05
DW (kg/plot)	−0.08	0.06	0.10		−0.16*	−0.01	−0.04	−0.06	0.00	0.03	0.02
GW (kg/plot)	0.14*	0.47**	0.50**	−0.04		−0.15*	0.07	0.06	0.07	0.01	−0.03
NDM (%)	0.02	−0.16*	−0.17*	−0.02	−0.29**		−0.28**	−0.15*	0.03	−0.02	0.13*
NDF (%)	−0.10	−0.08	−0.10	0.10	0.08	−0.42**		0.84**	0.63**	−0.75**	−0.81**
ADF (%)	−0.11	−0.14*	−0.13*	0.13*	−0.02	−0.29**	0.84**		0.76**	−0.90**	−0.91**
ADL (%)	−0.06	−0.10	−0.10	0.12*	−0.07	−0.17*	0.64**	0.67**		−0.81**	−0.79**
ME (MJ/kg DM)	0.11*	0.19**	0.21**	−0.06	0.15*	0.19**	−0.76**	−0.83**	−0.81**		0.97**
IVOMD (%)	0.09	0.11	0.12*	−0.08	0.03	0.32**	−0.81**	−0.81**	−0.82**	0.96**	

The color is proportional to negative (red) and positive (green) correlations, the diagonally down is control correlation values, and diagonally up is drought correlation values. DFL, days to fifty percent flowering; PH, plant height; FW, fresh weight; DW, dry weight; GW, grain weight; NDM, nitrogen on a dry matter basis; ADF and NDF, acid and neutral detergent fiber; ADL, acid detergent lignin; ME, metabolizable energy; IVOMD, in vitro organic matter digestibility; *significant at p < 0.05; **significant at p < 0.01.

### Linkage Map Construction

The marker coverage over the 10 sorghum chromosomes from raw and filtered data with 90% coverage is presented in Table 4. The average marker coverage was one marker per 2.22 cM, and the total map length of all linkage groups was 1269.9 cM. The total markers from the raw file with 321 entries are 292,960 and at 90% coverage, it is 1,141 for 314 entries. Out of 1,141 markers available for constructing the linkage map, 1,036 markers (after deleting the markers with position value 0) were mapped. The markers removed while grouping during linkage map construction are provided in Supplementary Table 1. The average linkage group (LG) length is 362 cM, with a minimum of 234.7 cM for S8 and a maximum of 507.7 for S10. The density of SNP markers per LG, on average, is 3.5 (lowest on S6-2.47 and highest on S10-4.89). A linkage map of 294 markers against respective positions has been provided in Supplementary Figure 2.

**TABLE 4:** Marker coverage across the 10 linkage groups in sorghum for ICSV1 × ICSV700, F7 recombinant inbred line population.

Linkage group	Data generated	SNPs for constructing genetic map	SNP generated	SNPs mapped	Length (cM)	SNPs/cM
S1	43,113	110	105	104	439.7	4.23
S2	36,501	113	101	101	345.9	3.43
S3	36,539	153	135	135	375.5	2.79
S4	31,910	117	106	106	354.9	3.35
S5	26,139	127	122	122	407.3	3.34
S6	26,798	114	106	105	259.2	2.47
S7	21,652	85	82	81	298.5	3.69
S8	21,553	109	77	77	234.7	3.05
S9	23,593	103	97	97	404.9	4.18
S10	25,162	110	105	104	507.7	4.89
Total	292,960	1,141	1,036	1,032	3,628.3	35.42

### QTL Analysis

**FIGURE 1:**
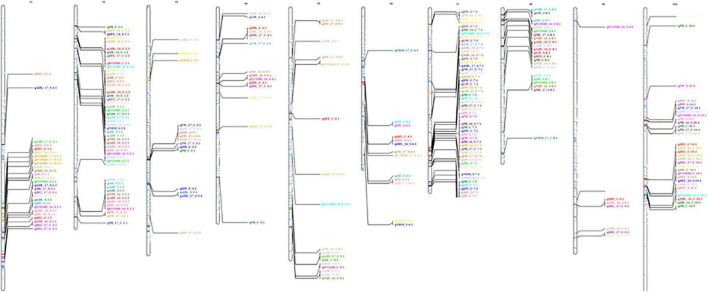
Graphical representation of linkage groups on chromosome for all 11 traits under control and treatment for 2 years.

A QTL analysis was performed using the BIP function of IciMapping (Meng et al., 2015) for all 11 traits under treatment and year-by-treatment (both control and drought), yielding 295 QTLs (Figure 1). Positive additive effect indicated a positive allele effect from the male parent (ICSV700) and a negative allele effect from the female parent (ICSV1). The complete data on significant QTL identified for 11 traits under 2 years and treatments have been provided in Supplementary Table 2.

### QTL Identification

**FIGURE 2:**
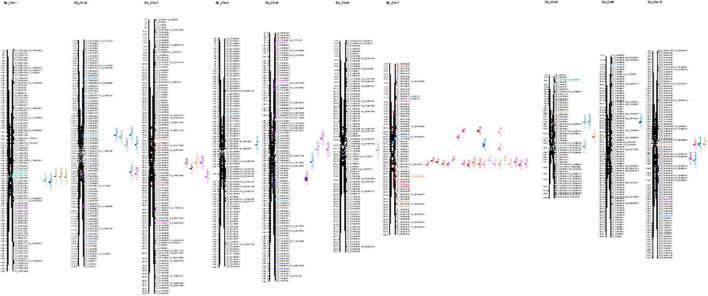
Graphical representation of linkage groups on chromosome for all dry weight and IVOMD under control and drought treatments for 2 years.

The current study identified 139 QTLs under control and 155 QTLs under drought across years and treatments. In the year 2016, a total of 91 QTLs were identified, 43 QTLs under control for ADF, ADL, DFL, DW, FW, GW, IVOMD, ME, NDF, PH, while 48 under drought for ADF, ADL, DFL, DW, FW, GW, IVOMD, ME, NDF, NDM, PH traits were identified. However, in year 2017, a total of 85 QTLs were mapped, 42 under control (ADF, ADL, DFL, DW, FW, GW, IVOMD, ME, NDF, NDM, and PH) and 43 (ADF, ADL, DFL, DW, FW, GW, IVOMD, ME, NDF, and PH) under drought. Across years under control, a total of 54 QTLs and, under drought, 64 QTLs were identified for all 11 traits in the present study, adding up to 118 QTLs (Figure 2 and Supplementary Table 2).

Out of 11 traits studied, the two prime traits—DW and IVOMD—have shown 35 and 24 QTLs (Table 5); DW showed 16 QTLs under control (6 QTLs across years, 4 QTLs in 2016, and 6 QTLs in 2017) and 19 QTLs under drought (6 QTLs across years, 5 QTLs in 2016, and 8 QTLs in 2017). Under control, significant QTL were identified for DW on LG7 (five QTLs), and on LG3 (one QTL), both positive and negative additive effects were mapped close to each other. For example, at position 231.96, there was an additive effect of 24.3 with 11.3% of variation and a LOD score of 8.4, while at position 260, there was a negative additive effect of—28.8 with 13% of variation and a LOD score of 12.8. Under drought, DW had 8 QTLs mapped, 6 on LG7 and one each on LG2 and 3, the highest additive effect was –33.06 with 10.4% variance explained and a LOD score of 6.7, whereas with an additive effect of –21.8, 15.3% variation was explained. IVOMD showed 9 QTLs under control (3 QTLs across years, 4 QTLs in 2016, and 2 QTLs in 2017) and 15 QTLs under drought (5 QTLs across years, 7 QTLs in 2016, and 3 QTLs in 2017). Under control, IVOMD mapped one QTL on LG2 with 7.44% PVE, 5.7 LOD value and 0.24 additive effects; likewise, IVOMD had 5 QTLs on LG1, 2 on LG2, and one on LG8. The maximum LOD 23.1 showed PVE of 3.94 (0.33 additive effect); maximum PVE was 7.09% with LOD of 5.5 and additive effect of 0.16.

**TABLE 5:** Quantitative trait loci detected for dry weight (DW) and digestibility (IVOMD) in ICSV1 × ICSV700 RIL population.

Trait	LG	Position	Left Marker	Right Marker	LOD	PVE (%)	Add*	Left CI	Right CI
**Control**									
DW	7	234.97	S7_54699104	S7_56368290	8.82	8.96	23.71	233.47	237.47
	7	254.97	S7_58694851	S7_59366675	6.84	7.15	21.09	254.47	255.47
	7	259.97	S7_59366675	S7_62915731	12.85	13.51	−28.85	259.47	262.47
**Drought**									
DW	7	229.97	S7_39535613	S7_32004530	4.83	8.76	16.52	228.47	230.47
	7	259.97	S7_59366675	S7_62915731	7.79	15.34	−21.87	258.47	263.47
IVOMD	1	353.99	S1_66894583	S1_65725731	6.06	5.32	−0.15	353.49	354.49
	8	17.54	S8_2392981	S8_3110973	5.51	7.1	0.17	16.04	19.04
**2016_Control**									
IVOMD	2	58.14	S2_58565145	S2_59425885	5.75	7.45	0.25	57.64	60.64
**2016_Drought**									
DW	7	229.97	S7_39535613	S7_32004530	5.44	7.8	28.53	228.47	230.47
	7	259.97	S7_59366675	S7_62915731	6.8	10.44	−33.02	258.47	263.47
IVOMD	1	361.99	S1_64479483	S1_64547158	6.17	5.65	−0.2	361.49	370.49
	2	120.14	S2_70384142	S2_71485265	4.54	4.48	0.17	117.64	121.64
	2	299.14	S2_43213849	S2_8884495	8.28	7.78	−0.23	294.64	300.64
**2017_Control**									
DW	3	228.09	S3_61565073	S3_61722531	7.4	9.44	22.15	224.59	229.59
	7	231.97	S7_32004530	S7_54709064	8.43	11.3	24.3	231.47	232.47
	7	259.97	S7_59366675	S7_62915731	8.42	11.43	−24.29	258.47	262.47
**2017_Drought**									
DW	2	306.14	S2_7659353	S2_4271638	5.77	6.51	16.77	303.64	310.64
	3	229.09	S3_61565073	S3_61722531	4.36	4.67	14.26	225.59	229.59
	7	243.97	S7_57555393	S7_57598884	5.00	8.24	−18.79	242.47	244.47
	7	259.97	S7_59366675	S7_62915731	5.05	5.3	−15.15	258.47	264.47
IVOMD	1	328.99	S1_74475248	S1_73811826	16.32	2.84	−0.29	328.49	329.49
	1	330.99	S1_73791442	S1_71687167	23.16	3.95	0.34	330.49	331.49
	1	334.99	S1_73791442	S1_71687167	6.22	1.45	−0.21	334.49	335.49

*QTL with positive effect is by alleles coming from the first parent (ICSV1), and QTL with negative effect is from the second parent (ICSV700).

### Epistasis QTLs

The total number of epistatic interactions across 11 traits was 534 with a min LOD value of 4.00 and maximum of 52.7 (Supplementary Figure 3 and Table 3). Upon sorting for LOD, more than 1,086 additives by additive epistatic effect of QTL at the two scanning positions were observed. The epistatic interactions for DW and IVOMD were analyzed (Table 6). Epistatic interactions were observed for DW under control for both years and under drought in year 2017. For IVOMD, the epistatic interactions were observed under control in 2016 and under drought in 2017. DW explained phenotypic variation of 36.21% with LG1, 5, 7, 8, 9, and 10 having two epistatic interactions, and IVOMD explained phenotypic variation of 18.66% with LG2, 3, 5, and 10 having two epistatic interactions. The values presented here are all above LOD 5, highest LOD = 6.8 between LG2 and 10, followed by LOD = 6.2 between LG2 and 3.

**TABLE 6:** Epistatic interactions for dry weight and the digestibility trait in ICSV1 × ICSV700 RIL population.

Trait	LG	Total interactions	LGs in interaction*	PVE range
Dry weight	1	12	**1**, 3, 6, 7, 8, 9, and 10	1.1–6.4
	2	5	**5, 6**, 7, and 10	0.7–5.7
	3	4	5, 7, and 10	0.8–1.8
	4	4	4, 5, **7**, and 10	1.2–4.8
	5	4	5, 6, 7, and 10	0.6–2.6
	6	4	6, **7,** 9, and **10**	2.5–6.7
	7	4	7, 8, and **10**	1.6–5.6
	9	3	9 and 10	3.3–4.1
	10	4	10	1.8–4.5
IVOMD	1	3	5, **7**, and **9**	2.0–7.8
	2	8	3, 4, 5, 7, 9, and **10**	1.5–7.2
	3	6	3, 4, 5, **6**, 9, and 10	1.5–9.2
	4	4	**4** and 6	1.4–4.1
	5	4	5 and **10**	1.5–7.3
	6	2	**6** and 8	2.9–5.0
	7	2	9	4.6–6.7
	10	1	10	1.92

*Bold shows a PVE% of ≥4.

### Identification of Genomic Regions and Gene Annotations

**FIGURE 3:**
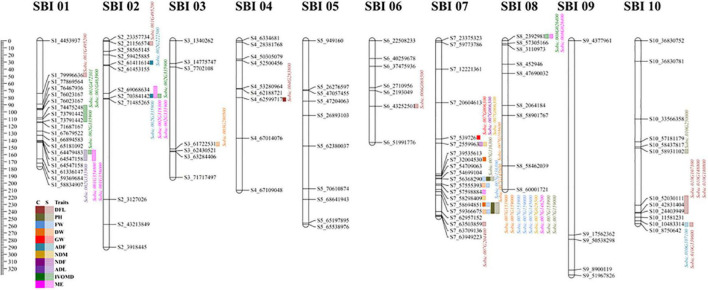
Graphical representation of linkage groups on chromosome for all 11 traits under control and drought treatment for 2 years. The ruler in the left margin has been provided in cm next to the legend for all traits; the traits against which the gene is mapped are color coded; solid color indicates control, and faded color indicates drought. Gene annotation for specific marker was performed using Phytozome 12.1.6.

A total of 294 QTLs were identified for 11 traits under two treatments and years; the DNA sequences underlying these QTL were blasted against the *Sorghum bicolor* sequence. This provided information on gene, mRNA and CDS, and respective gene ids for QTLs (Supplementary Table 4). Multiple traits have similar gene ids to avoid repetition, a unique list of gene ids was annotated using Phytozome 12.1.6 yielding function/pathway of the gene involved (Supplementary Table 5). These QTLs were then blasted using sequence of *Sorghum bicolor* ver. 3.1 in Phytozome, yielding 315 gene ids for 11 traits under control and drought across both years. Out of these, duplicated genes were removed to identify a unique set, resulting in 42 genes. These 42 gene ids were annotated and plotted over linkage groups (Figure 3).

The gene id *Sobic.001G495200* identified for flowering under drought condition was mapped on LG1 at 76463309, which has oxidoreductase activity that belongs to family disulfide oxidoreductase and involved in glutathione metabolism. A gene (*Sobic.001G438800* on LG1 at 71687096) related with nitrogen content (on a dry matter basis) under drought was related with a beta catenin-related armadillo repeat with protein binding function on LG1. Another gene *Sobic.001G356000* had MYB-like DNA binding protein; this was mapped against the lignin content and metabolizable energy under drought conditions at 64546335 on LG 1, and N-terminal acetyltransferase was linked with *Sobic.001G463900*, which was associated with *IVOMD* under drought on LG 1 at 73790038.

A gene (*Sobic.002G223200*) comes under the protease family related to S26 mitochondrial inner membrane protease, which was located on LG2 at 61451145, belongs to the protein export pathway; this was found related to neutral detergent fiber under drought. Oxidoreductase gene (*Sobic.002G137800 on LG 2 at 21153582*) for flowering under drought and import in beta gene (*Sobic.002G350600 on LG2 at 71475963*) related to neutral detergent fiber under drought was also identified. A cyclin-related gene (*Sobic.002G335900 on LG2 at 70382979*) associated with nitrogen, metabolizable energy, and IVOMD under drought were found on LG2, and, additionally, acid detergent fiber was also tied to the same gene. A gene involved in the steroid biosynthesis pathway with methyltransferase function was identified (*Sobic.002G073700 on LG2 at 7653306*) for fresh and DW. On LG3, a gene with ATP binding (*Sobic.003G248600 at 58746137*), TP binding (*Sobic.003G280500 at 61563437*), and protein phosphorylation gene (*Sobic.003G291700 at 62426982*) were documented for 100 seed weight and DW. The gene *Sobic.003G248600* on LG3 at 58746137 is known to be involved in the proteasome pathway, mapped for test weight under drought. Another methyltransferase gene (*Sobic.004G283800 at 62599054*) and *Sobic.004G339100 at 67104883*, involved in diphthamide biosynthesis, were identified for flowering and GW under drought on LG4. Gene *Sobic.006G069500* at 43250944 with multiple functions, such as electron carrier activity, protein disulfide oxidoreductase activity, and cell redox homeostasis, belong to family glutaredoxin was on LG6, which was mapped for flowering under drought. Furthermore, a gene (*Sobic.006G162200 at 51991334*) with oxidoreductase activity, acting on NAD(P)H, quinone or a similar compound as an acceptor involved in the oxidation-reduction process, was found on LG6 for test weight.

A gene (*Sobic.007G153900 at 58690766 in LG 7*) involved in the nitrogen metabolism pathway related to NADH-cytochrome B5 reductase was linked to plant height and fresh and DW under both treatments. *Sobic.007G151100* gene on LG7 at 58295202 was involved in alpha-linolenic acid metabolism and in the current study mapped for nitrogen content under drought. A key identification was associated with starch and sucrose metabolism and pentose and glucoronate interconversions with gene *Sobic.007G146200* on LG7 at 57595992 mapped to metabolizable energy in the current study. DNA binding and regulation of transcription gene were located on LG 7 (*Sobic.007G136300 at 31999643*) for fresh weight and plant height under both treatments. On LG10, gene *Sobic.010G139600* (for flowering under drought) was identified with protein kinase activity, ATP binding, and was involved in protein phosphorylation, which belongs to the family mitogen-activated kinase. A gene belonging to plant pathogen interaction (*Sobic.010G148800 at 42829856*- for flowering under drought) and the ribosomal pathway (*Sobic.010G250000 at 58928662*) was also identified on LG10 for plant height.

**FIGURE 4:**
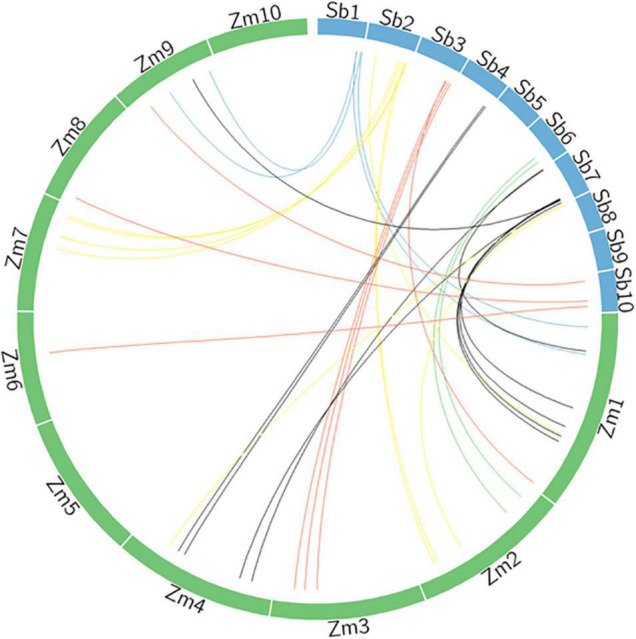
Syntenic relationship of sorghum genomic regions associated for traits under study with maize genome.

The analysis of the syntenic relationship between maize and sorghum for identified genes, with a sequence similarity of 98% or higher, is presented in Figure 4 (Supplementary Table 6). There are a number of maize orthologs for sorghum genes identified in the current study. Orthologous genes associated with both fresh and DW in sorghum, annotated on LG3 and 7, have been found on maize LG1, 4, and 9. Likewise, genes associated with digestibility (ME and IVOMD) have been mapped on to the sorghum LG1, 2, and 8 in this study and on to LG1, 2, 7, and 9 in maize. A gene associated with nitrogen content mapped onto LG7 in sorghum was mapped onto LG1 in maize (information from Phytozome V 12). The Sobic.001G438800 has shown 95.5% similarity for nitrogen content in maize on LG9; Sobic.001G495200 has 97.3% similarity with flowering and test weight under drought on LG 1 in maize. The Sobic.002G223200 for neutral detergent fiber mapped on LG 7 in maize showed 95.5% similarity index. Another gene, Sobic.002G318300, linked to both metabolizable energy and test weight showed 96.2% similarity with maize on LG2 at position 203912110-203915281. The fresh and DW mapped to Sobic.007G145600 under drought displayed 95.5% sequence similarity on LG1 in maize. About 98% similarity was shown by Sobic.007G151100 for gene mapped on maize at LG 1 for nitrogen content on a dry matter basis. The gene Sobic.007G153900, mapped to multiple traits in sorghum (plant height under drought and control, fresh weight under drought, DW under control across years), was linked on LG4 in maize with 95.7% similarity.

## Discussion

Extensive mapping is essential for crop improvement by undermining yield and quality. One of the major conclusions drawn for sorghum is that the opportunities for marker-assisted breeding for fodder quality and nutrition exist, but these are not mapped (Hash et al., 2003). QTL mapping for fodder quality traits has not been reported so far. The closest study reported is the association study performed in sorghum for 5 fodder quality traits by identifying 42 SNPs (Li et al., 2018) and population genomic study performed for lignin content in forage sorghum (Niu et al., 2020).

### Agronomic and Fodder Quality Under Drought

The genetic dissection of quantitatively inherited traits is essential to improve the selection efficiency. It can be enhanced by indirect selection of associated traits (Habyarimana et al., 2020). The agronomic traits have shown significant differences under treatment in both years. However, the fodder quality traits showed significant differences under drought in 2017 and high mean parent comparison across population and parents in 2016. Thus, concomitant improvement of agronomic and fodder traits by exploiting cultivar-dependent variations will focus on targeted genetic enhancement (Aruna et al., 2015; Blümmel et al., 2015). DW was significantly correlated with fresh weight and plant height. Although the intensity is less, the positive significance indicated the population under study has a natural variation for biomass yield and its associated traits. Significant correlation also has been reported between biomass yield and plant height (Mathew et al., 2018; Habyarimana et al., 2020). Nonetheless, fiber fractions and digestibility traits have shown significantly negative correlation, yet no negative significant correlation between grain and biomass was observed against digestibility traits. This indicates that the breeding can be targeted for simultaneous improvement of biomass yield and digestibility traits with no tradeoff for grain and stover content (Aruna et al., 2015; Blümmel et al., 2015; Somegowda et al., 2020).

### Significant QTLs Mapped

The QTL map generated 35 significant loci for DW (on LG2, LG3, LG5, LG7, LG8, and LG10), 29 for fresh weight (on LG2, LG3, LG5, LG6, LG7, and LG10), and 42 for plant height (on all LG except 5), followed by 51 flowering (on all LG). This shows that main and associated traits are being tightly influenced. Plant height and DW yield has hotspot on LG 7 (Shiringani and Friedt, 2011; Upadhyaya et al., 2012; Habyarimana et al., 2020). Another QTL for plant height reported by Bai et al. (2017) was *sam10667-sam41751* located on LG7. The plant height genes Sobic.*003G202000*, *Sobic*.*008G146100, and Sobic*.*009G207100* were identified and reported by Luo et al. (2020) on LG3, 8, and 9. Plant height has been mapped on LG1, 7, and 9 for bioenergy-related traits in sorghum (Guan et al., 2011). Plant height QTL *qHT7.1* and *Dw3* identified through linkage mapping, on LG7 and *Dw1* on LG9, are known to modify gene expressions for high biomass and lodging resistance through optimizing plant height (Li et al., 2015). Similarly, 14 QTL on LG9, 8 on LG7, 2 on LG8, and one on LG2 were identified for plant height in sorghum (Girma et al., 2019). On LG2, 3, 6, and 9, QTL for plant height and on LG3, 7, and 8, and QTL for biomass yield (*qBY-7* with flanking marker S7_57890877–S7_58551650), were reported by Gelli et al. (2017). Many QTLs on LG1, 3, 5, 6, 8, 9, and 10 for biomass yield were reported in response to water availability (Phuong et al., 2019). A potential candidate gene for biomass yield 1 (*BY1*-mutant), *Sobic.002G379600* of LG 2 (73572791-73577695), has been reported (Chen et al., 2020). *qTB4*, *qTB7.1*, and *qTB7.2* on LG4 and 7, respectively, were reported for total biomass with gene id *Sobic.007G005400*, *Sobic.007005500*, and several association studies reported significant SNPs on LG7 for flowering (Moghimi et al., 2019). One QTL on LG6 for flowering and plant height has been observed, this could be positive confirmation for associated biomass yield traits, as a *NAC* gene has been reported on LG6 that has variation in biomass properties and yield potential (Xia et al., 2018). Fine mapping on LG10 has identified QTL for *stg* (a drought-associated trait); however, in the current study, we found flowering, plant height, and acid detergent fiber on LG10. Thirteen QTLs on LG 2, 3, 6, 7, 8, and 9, out of which five QTLs for days to heading, three for plant height and total shoot fresh weight and two for Brix, were reported by Kajiya-Kanegae et al. (2020). QTLs for flowering time were identified on LG2, 6, and 9 by Sukumaran et al. (2016), along with Stay-green (*stg*) QTLs in LG3 and 4.

The LG6 was fine mapped for dry stalk yield with allelic variation for four genes, contributing to increased stalk mass, and higher biomass digestibility in sorghum (Xia et al., 2018). This supports the current results that three QTL clusters, each one for flowering, plant height, and fresh weight, were mapped on LG6. An intermodal length QTL (*Dw07_55.1*) has been mapped on LG7 (Hilley et al., 2016). A SNP for associated trait like stem circumference (S7_59261924) was known to overlap each other in the known QTL region that starts at 59450000 and ends at 59550000 (Zhao et al., 2016). Another derivative traits for plant height – culm length was mapped on LG7, in the same region as the *dw3* gene for plant height (Shehzad and Okuno, 2015).

Among the fodder quality traits, metabolizable energy and nitrogen content have 1 QTL mapped on LG7. An acid detergent fraction showed four QTLs on LG2 and one on LG10; neutral detergent fiber has QTL on LG1, 2, 5, 7, 8, and lignin (ADL) had 5 QTL on LG 1 and 2. Digestibility and metabolizable energy were mapped on LG1, 2, 7, and 8. This is in accordance with crude protein quantitative trait nucleotide (QTN) reported on LG7 (Li et al., 2018). Fiber fraction (NDF) was mapped on LG5 in the current study, and as reported by Li et al. (2018), crude protein and NDF and ADF had one QTN on LG5. GT47 proteins participate in xylan and xyloglucan biosynthesis in plants, and 39 sorghum GT47 genes have been reported on LG1, 2, 3, 4, 6, 7, 8, 9, and 10 (Xu et al., 2018). A total of 27 laccase candidates (*SbLAC1-SbLAC27*) were identified on LG1, 2, 3, 4, 5, 8, 9, and 10 (Wang et al., 2017). Genes in the lignin biosynthesis pathway were located on SBI-04 and SBI-07 (two genes each), and another five genes on SBI-04 were co-localized with genes for stem composition (Wang et al., 2020). Furthermore, drought-induced lodging in grain sorghum is known to be associated with plant height and traits linked to carbon remobilization (Wang et al., 2020). Population genomic and genome-wide association analysis of lignin content in a global collection of 206 forage sorghum accessions showed significant association on LG1, 4, 6, 7, and 9 (Niu et al., 2020). Two significant SNPs reported for starch are located on LG1 and 3; however, starch SNPs were distributed across LG1, 3, 4, 5, 6, 9, and 10 (Chen et al., 2019). The current study confirms QTL for starch-attributed traits (fiber fractions) on LG2, 3, 5, 8, 9, and 10. Additionally, genes for nitrogen (*Sobic.002G335900* at 70382979-70385365), fiber fractions (acid- *Sobic.002G335900* at 70382979-70385365 and neutral- *Sobic.002G350600* at 71475963-71485759), ME (*Sobic.002G318300* at 69068162-69076442), and IVOMD (*Sobic.002G335900* at 70382979-70385365) were identified.

Two important studies from the analogous crop for fodder quality were reported by Vinay et al. (2013) and Wang et al. (2016) in maize. Ten significant genomic regions for nitrogen content were reported in maize on LG1, 3, 5, 7, 8, and 9 (Vinay et al., 2013). In another study, 73, 41, and 82 SNPs were found to be associated with ADF, NDF, and (*in vitro* dry matter digestibility) IVDMD, respectively, in maize (Wang et al., 2016). In the current study, nitrogen content was found on LG2, 3, 6, 7, and 8 with highest LOD of 10.57 on LG7, while, for IVOMD on Chromosomes 1, 3, 4, 5, 6, and 9 for maize and in sorghum out of 24 QTLs identified 2 QTLs on LG1 S1_74475248 and S1_73791442 on position 328 (LOD > 16) and 330 (LOD > 23), respectively.

### Genomic Regions and Annotations

A study reported similar results for *Sobic.001G495200*, *Sobic.007G153900*, and *Sobic.007G159000* identified for plant height under salinity (Wang et al., 2020), while *Sobic.002G261500* (the auxin-related family), *Sobic.002G223200* (peptidase-family protein), *Sobic.002G137800* (a putative protein), *Sobic.002G188200* (similar to the ring finger), and an Erwinia-induced protein (*Sobic.002G222500*) for above ground biomass were reported by Hostetler et al. (2020). A *Sobic.004G283800* gene, similar to Dehydration-responsive family protein, was reported for biomass under salinity and control (Hostetler et al., 2020; Wang et al., 2020). Dry Stalk (D) locus with a NAC transcription factor (*Sobic.006G147400*) with a stop codon has been reported (Xia et al., 2018). In sorghum, fodder quality traits were studied using genome-wide association, which reported crude protein (*Sobic.002G217100*, *Sobic.002G397001*), cellulose-NDF fiber fraction (*Sobic.002G390800*), and NDF (*Sobic.002G390800*) genes on LG2 (Somegowda et al., 2021). Additionally, *Sobic.001G095700* and *Sobic.009G132000* were also reported for hemicellulose (ADF fiber fraction) (Li et al., 2018). The synteny studied between agronomic and fodder traits for sorghum and maize will prove resourceful for predicting the position of genes, conferring key traits.

### Epistatic Interactions

The agronomic traits discussed above and the fodder quality traits are all quantitative in nature, owing to genetic variability and additive × additive epistatic interactions, which confirm that the trait was governed by one or two major genes (Yadav et al., 2019). The DW qDW_C-3-1 corresponding to *Sobic.003G280500*, likewise for IVOMD, qIVOMD_S-2-1, qIVOMD_S-2-2, and qIVOMD_C-2-1 on LG2 correspond to *Sobic.002G335900*. Although it is a quantitative trait, here, it indicates that it is the major contributing gene in the current population.

## Conclusion

The agronomic and fodder quality traits in terms of biomass and GW have shown no trade off toward nitrogen content and digestibility neither under control nor under drought in the current study. This indicates that the concurrent improvement of these traits is plausible. Another key finding is mapping of known gene *Sobic.001G356000* for lignin content and metabolizable energy under drought conditions and gene *Sobic.001G463900* for IVOMD under drought conditions. The epistatic interactions have also been observed for DW and IVOMD on LG10. Thus, it is a positive indication that breeding efforts can be channelized to improve these traits concomitantly. We show synteny between maize and sorghum with high sequence similarity for agronomic and fodder traits. These results can be further confirmed using fine mapping and *in silico* validation for sorghum.

## Data Availability Statement

The datasets presented in this study can be found in online repositories. The names of the repository/repositories and accession number(s) can be found below: https://doi.org/10.21421/D2/BM3HI1 (phenotypic data) and https://doi.org/10.21421/D2/3UKRCK (genotypic data).

## Author Contributions

SD, RG, and VS: conceptualization. SD, AR, and VS: methodology. VS, AV, KP, and SS: formal analysis and investigation. VS: writing—original draft preparation. All authors review and editing. AR, SD, RG, and CJ: funding acquisition and resources. SD, RG, CJ, and JN: supervision.

## Conflict of Interest

The authors declare that the research was conducted in the absence of any commercial or financial relationships that could be construed as a potential conflict of interest.

## Publisher’s Note

All claims expressed in this article are solely those of the authors and do not necessarily represent those of their affiliated organizations, or those of the publisher, the editors and the reviewers. Any product that may be evaluated in this article, or claim that may be made by its manufacturer, is not guaranteed or endorsed by the publisher.

## Footnotes

1
https://www.fao.org/faostat/en/#data/QCL

2
http://idc.icrisat.org/idc/wp-content/uploads/2019/01/Final-Green-fodder-production_Booklet.pdf

3
https://phytozome-next.jgi.doe.gov/info/Sbicolor_v3_1_1

4
www.isbreeding.net
