# Supplementary material for: Genetic Dissection and Quantitative Trait Loci Mapping of Agronomic and Fodder Quality Traits in Sorghum Under Different Water Regimes
Source: Front Plant Sci. 2022 Feb 17;13:810632. doi: 10.3389/fpls.2022.810632 (PMC8892184; doi:10.3389/fpls.2022.810632)
Supplement: Supplementary file 1 [file Presentation_1.PPTX]

## Slide 1
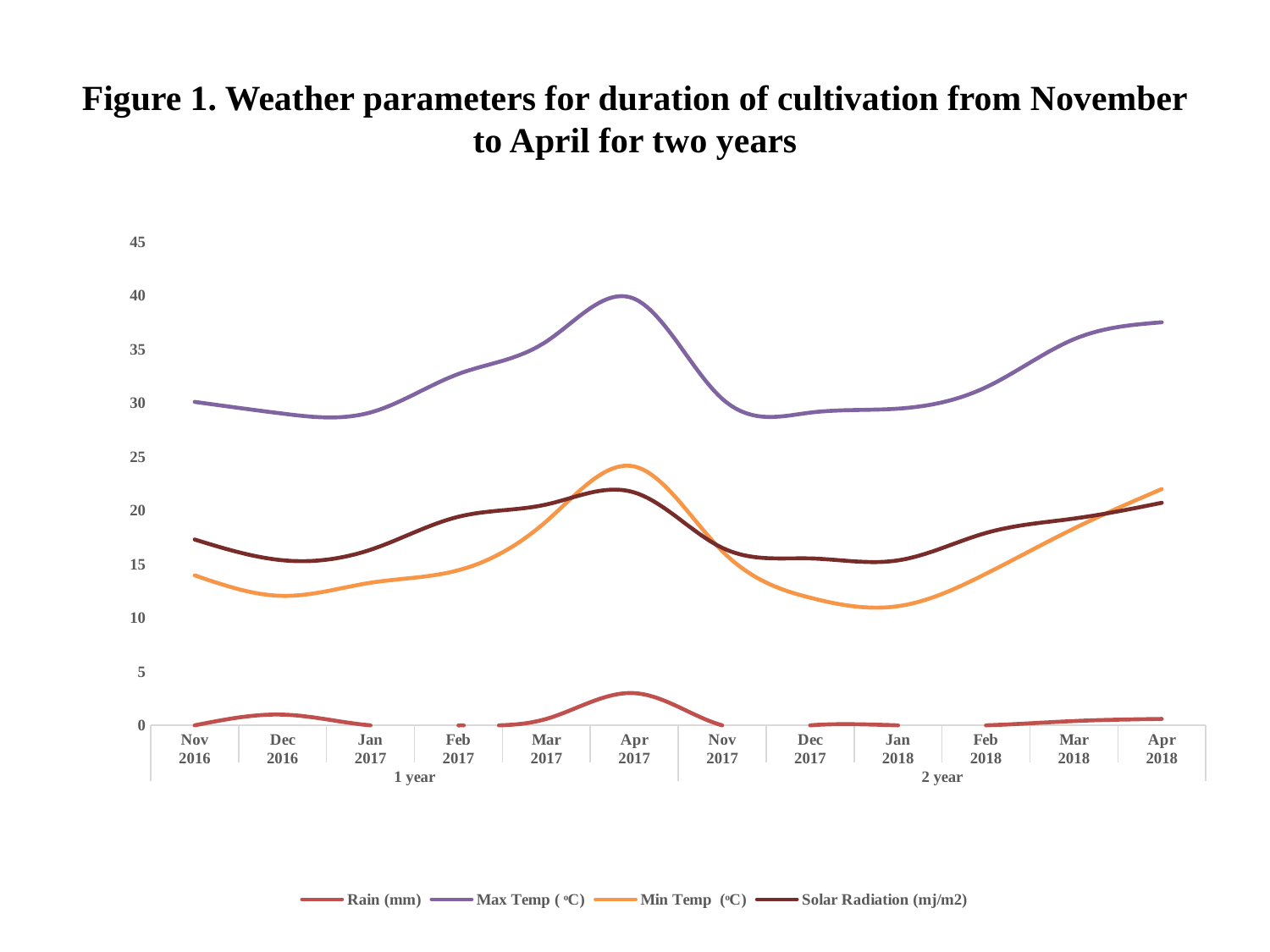

# Figure 1. Weather parameters for duration of cultivation from November to April for two years
### Chart
| Category | Rain (mm) | Max Temp ( ᵒC) | Min Temp  (ᵒC) | Solar Radiation (mj/m2) |
|---|---|---|---|---|
| Nov | 0.0 | 30.11 | 13.96 | 17.3 |
| Dec | 1.0 | 29.02 | 12.05 | 15.37 |
| Jan | 0.0 | 29.13 | 13.27 | 16.35 |
| Feb | 0.0 | 32.71 | 14.44 | 19.42 |
| Mar | 0.6 | 35.73 | 19.0 | 20.56 |
| Apr | 3.0 | 39.71 | 24.1 | 21.68 |
| Nov | 0.0 | 30.4 | 16.22 | 16.53 |
| Dec | 0.0 | 29.11 | 11.88 | 15.54 |
| Jan | 0.0 | 29.48 | 11.09 | 15.36 |
| Feb | 0.0 | 31.47 | 14.11 | 17.91 |
| Mar | 0.4 | 35.94 | 18.32 | 19.25 |
| Apr | 0.6 | 37.52 | 22.0 | 20.72 |

## Slide 2
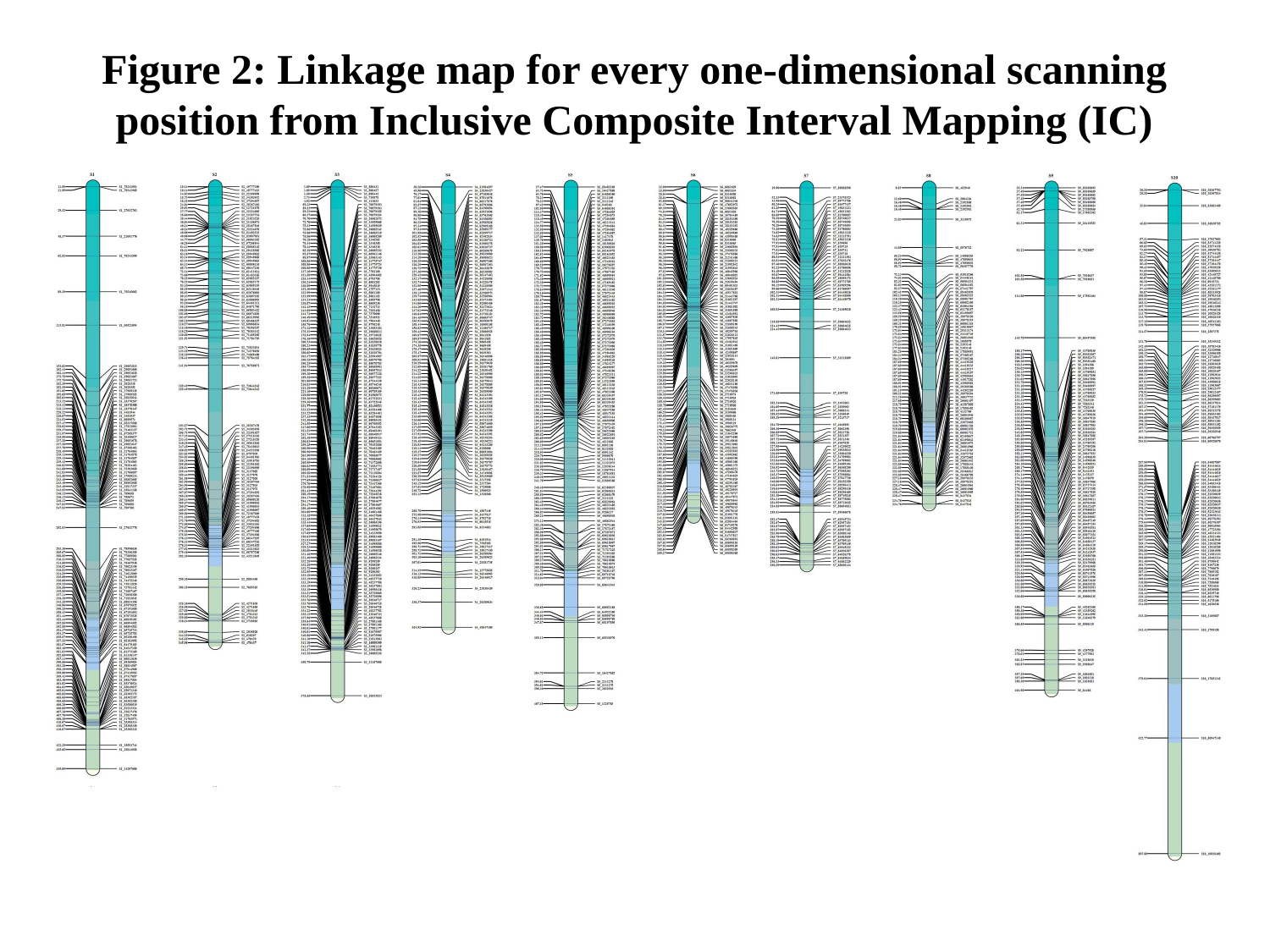

# Figure 2: Linkage map for every one-dimensional scanning position from Inclusive Composite Interval Mapping (IC)

## Slide 3
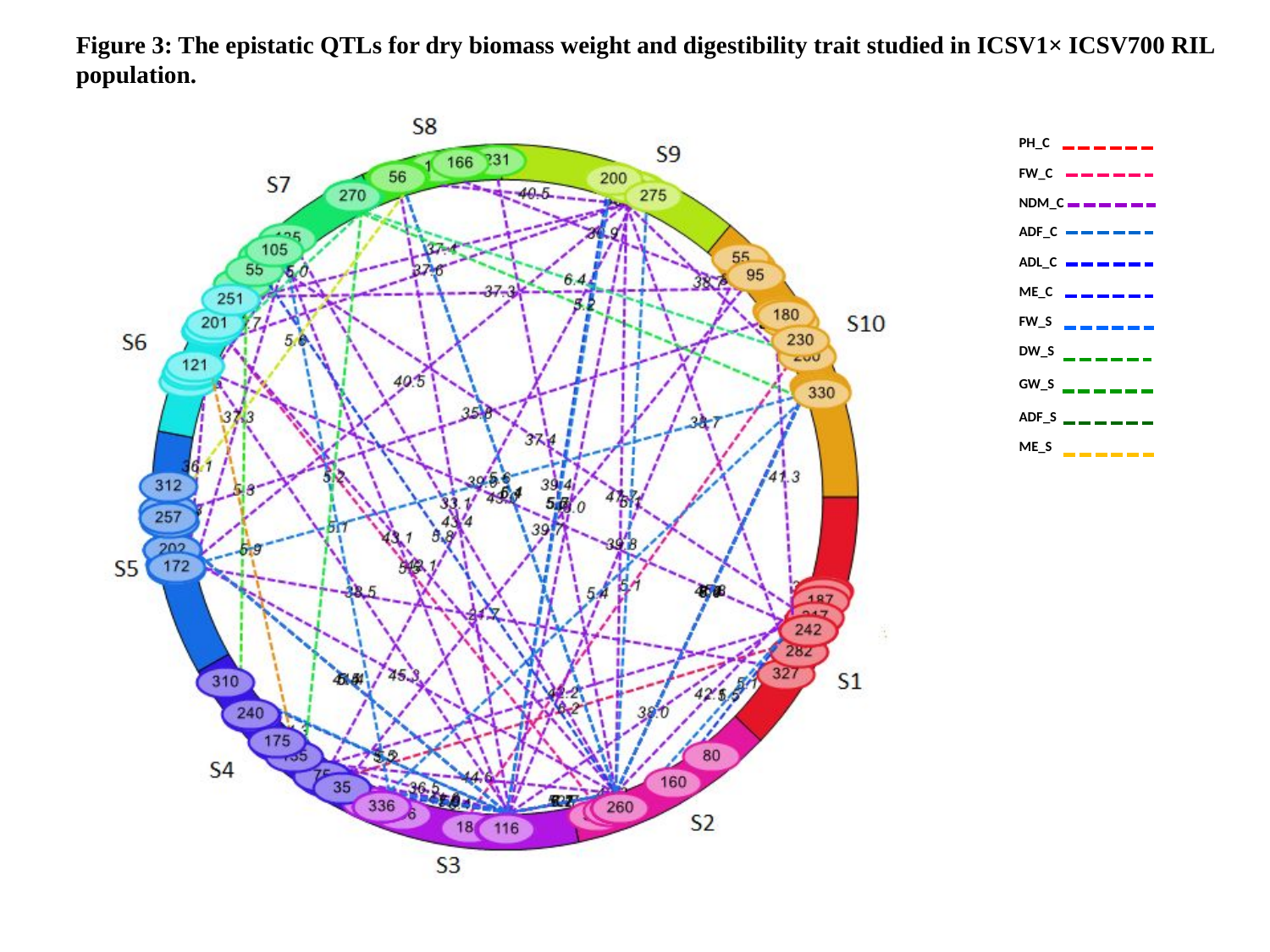

Figure 3: The epistatic QTLs for dry biomass weight and digestibility trait studied in ICSV1× ICSV700 RIL population.
| PH\_C |
| --- |
| FW\_C |
| NDM\_C |
| ADF\_C |
| ADL\_C |
| ME\_C |
| FW\_S |
| DW\_S |
| GW\_S |
| ADF\_S |
| ME\_S |
